# Supplementary material for: The assembly of microbial communities on red sandstone surfaces was shaped by dispersal limitation and heterogeneous selection
Source: mSystems. 2025 Dec 19;11(1):e01600-25. doi: 10.1128/msystems.01600-25 (PMC12817949; doi:10.1128/msystems.01600-25)
Supplement: Supplemental Figures — Figures S1 to S3. [file msystems.01600-25-s0001.docx]

**Supplementary Figures**


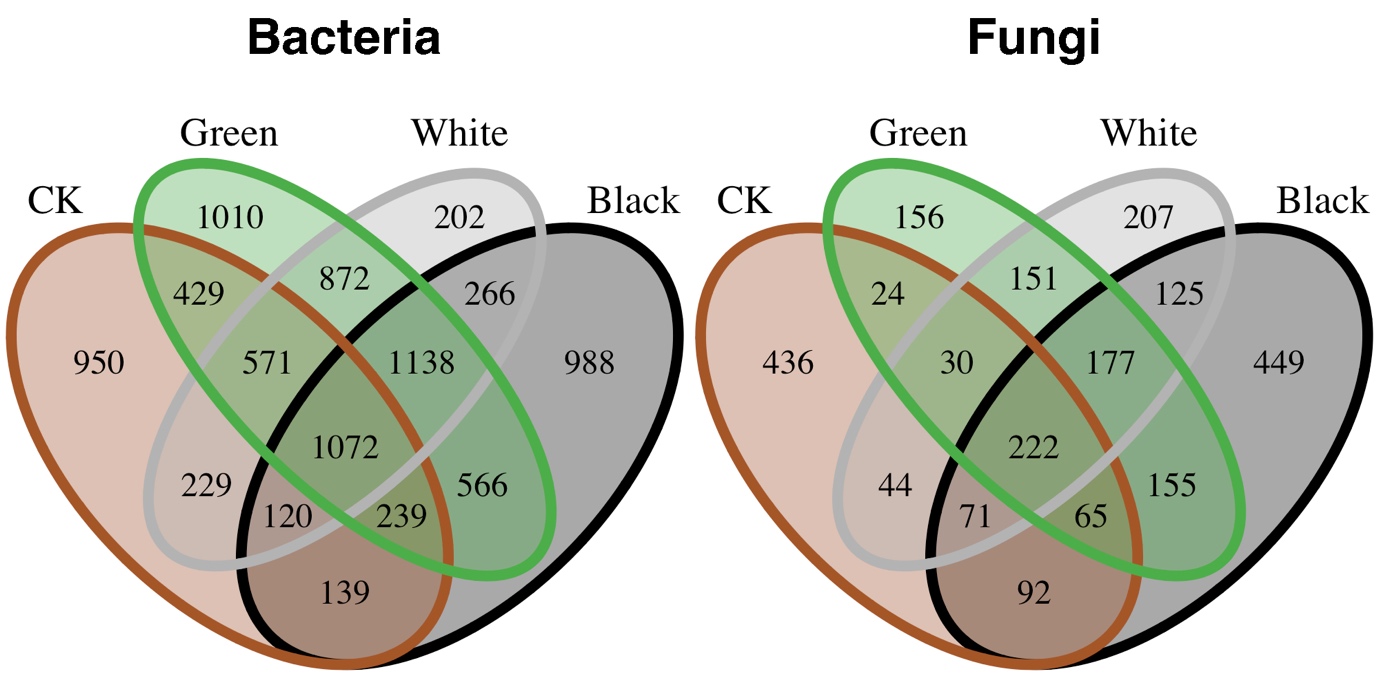


**Figure S1.** The overall taxonomic community composition among all the samples. (a). The overall taxonomic community composition of the bacteria. (b). The overall taxonomic community composition of the fungi.


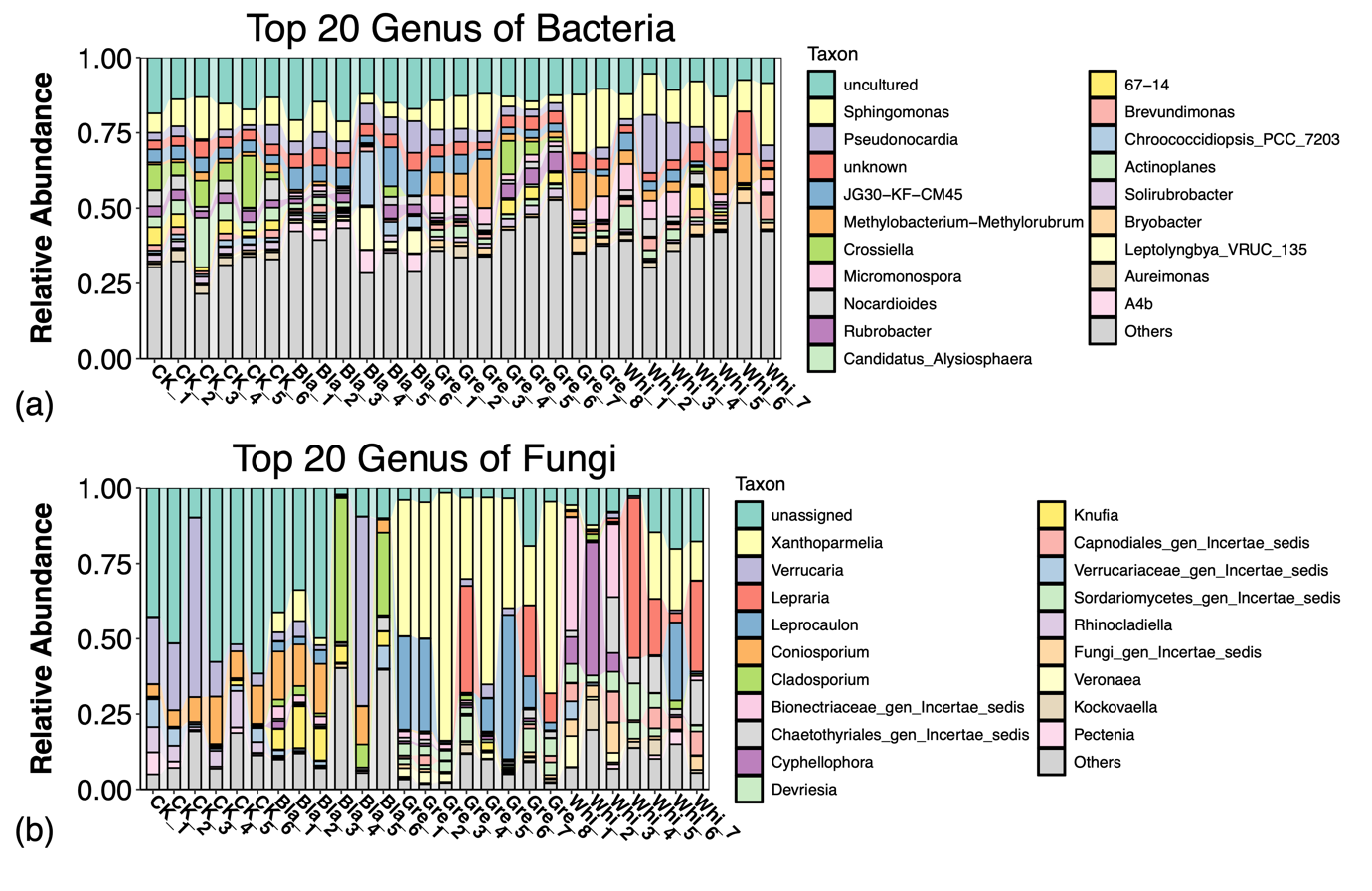


**Figure S2.** Taxonomic community composition of the microbial communities in genus level. (a). Relative abundances of dominant bacteria among 27 samples in genus level. (b). Relative abundances of dominant fungi among 27 samples in genus level.


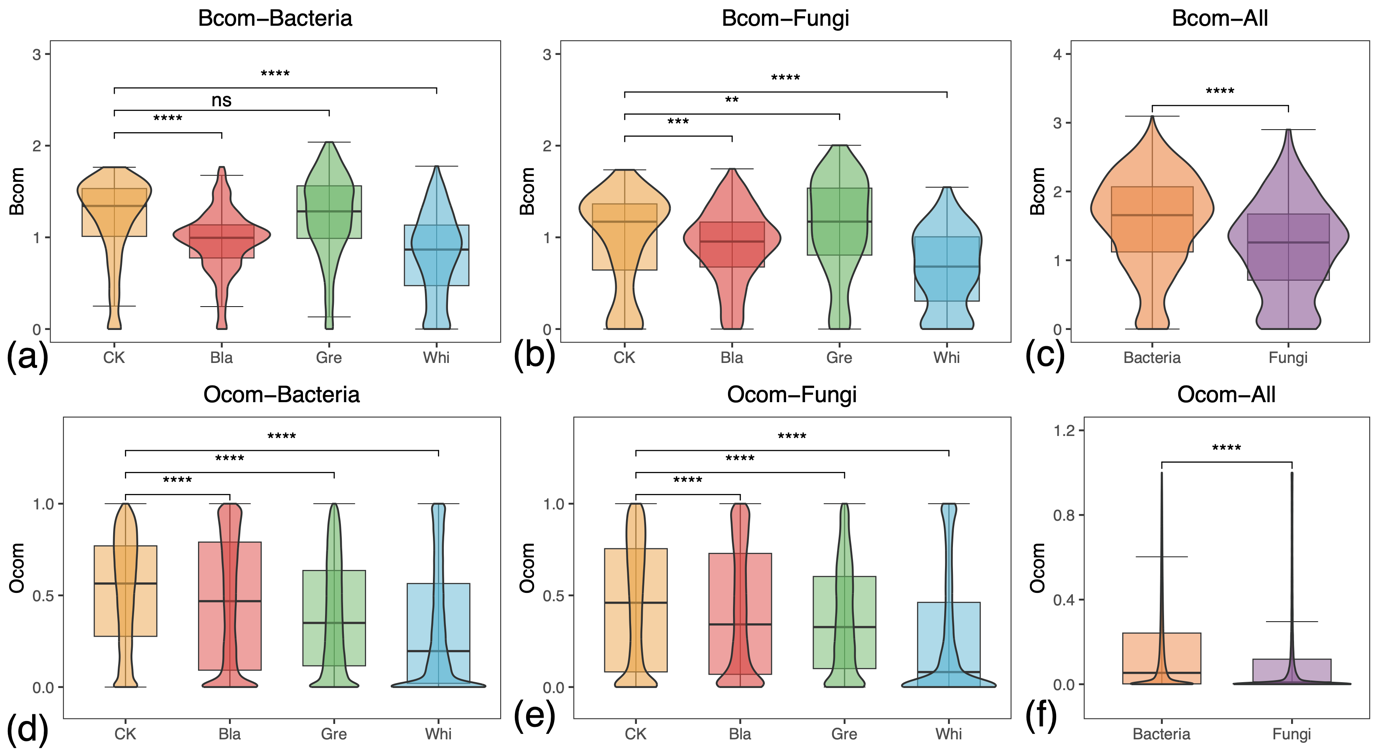


**Figure S3.** Niche characterization of the stone surface microbial communities based on Shannon niche breadth index and Pianka niche overlap index. (a). Wilcox test illustrating the average habitat niche breadth (*Bcom*) differences among three groups of bacteria samples comparing with CK in bacteria based on Shannon index. (b). Wilcox test illustrating the average *Bcom* differences among three groups of bacteria samples comparing with CK in fungi based on Shannon index. (c). Wilcox test illustrating the average *Bcom* differences between bacteria and fungi based on Shannon index. (d). Wilcox test illustrating the average habitat niche overlap (*Ocom*) differences among three groups of bacteria samples comparing with CK in fungi based on Pianka niche overlap index. (e). Wilcox test illustrating the average *Ocom* differences among three groups of bacteria samples comparing with CK in fungi based on Pianka niche overlap index. (f). Wilcox test illustrating the average *Ocom* differences between bacteria and fungi based on Pianka niche overlap index.
