# Supplementary material for: The assembly of microbial communities on red sandstone surfaces was shaped by dispersal limitation and heterogeneous selection
Source: mSystems. 2025 Dec 19;11(1):e01600-25. doi: 10.1128/msystems.01600-25 (PMC12817949; doi:10.1128/msystems.01600-25)
Supplement: Captions — for supplemental tables. [file msystems.01600-25-s0002.docx]

**Legends for supplementary tables**

**Table S1.** Relative abundances of dominant bacteria among 27 samples at the phylum level.

**Table S2.** Relative abundances of dominant bacteria among 27 samples at the genus level.

**Table S3.** Relative abundances of dominant fungi among 27 samples at the phylum level.

**Table S4.** Relative abundances of dominant fungi among 27 samples at the genus level.

**Table S5.** Habitat niche breadth of bacteria at the phylum level.

**Table S6.** Habitat niche breadth of fungi at the phylum level.

**Table S7.** Summaries for the details from the metagenome sequencing
